# Molecular pathology of vertebral deformities in hyperthermic Atlantic salmon (*Salmo salar*)

**DOI:** 10.1186/1472-6793-10-12

**Published:** 2010-07-06

**Authors:** Elisabeth Ytteborg, Grete Baeverfjord, Jacob Torgersen, Kirsti Hjelde, Harald Takle

**Affiliations:** 1 Nofima Marin, Norwegian Institute of Food, Fisheries and Aquaculture Research, P.O. Box 5010, NO-1432 Ås, Norway; 2 Norwegian University of Life Sciences, NO-1432 Ås, Norway; 3 AVS Chile SA, Imperial 0655, Of. 3A, Puerto Varas, Chile

## Abstract

**Background:**

Hyperthermia has been shown in a number of organisms to induce developmental defects as a result of changes in cell proliferation, differentiation and gene expression. In spite of this, salmon aquaculture commonly uses high water temperature to speed up developmental rate in intensive production systems, resulting in an increased frequency of skeletal deformities. In order to study the molecular pathology of vertebral deformities, Atlantic salmon was subjected to hyperthermic conditions from fertilization until after the juvenile stage.

**Results:**

Fish exposed to the high temperature regime showed a markedly higher growth rate and a significant higher percentage of deformities in the spinal column than fish reared at low temperatures. By analyzing phenotypically normal spinal columns from the two temperature regimes, we found that the increased risk of developing vertebral deformities was linked to an altered gene transcription. In particular, down-regulation of extracellular matrix (ECM) genes such as *col1a1*, *osteocalcin*, *osteonectin *and *decorin*, indicated that maturation and mineralization of osteoblasts were restrained. Moreover, histological staining and *in situ *hybridization visualized areas with distorted chondrocytes and an increased population of hypertrophic cells. These findings were further confirmed by an up-regulation of *mef2c *and *col10a*, genes involved in chondrocyte hypertrophy.

**Conclusion:**

The presented data strongly indicates that temperature induced fast growth is severely affecting gene transcription in osteoblasts and chondrocytes; hence change in the vertebral tissue structure and composition. A disrupted bone and cartilage production was detected, which most likely is involved in the higher rate of deformities developed in the high intensive group. Our results are of basic interest for bone metabolism and contribute to the understanding of the mechanisms involved in development of temperature induced vertebral pathology. The findings may further conduce to future molecular tools for assessing fish welfare in practical farming.

## Background

Industrial fish farming makes use of intensive production regimes in an effort to decrease production time and costs. Elevated water temperatures are commonly applied, often without explicit control of factors like nutrition, water quality, densities and vaccination. The intensive rearing systems are unfortunately correlated with deformities affecting both skeletal and soft tissues [1,2]. In teleosts, hyperthermia can induce vertebral deformities both during the embryonic development and after the vertebral column has been established [3-5]

The teleost vertebral body is built using a minimal bone mass to reduce negative buoyancy [6]. In salmon, the vertebral body comprises four mineralized or ossified layers. Formation of the different layers involves the balanced and highly regulated formation of bone and cartilaginous structures through patterns of mineralization and matrix deposition [7]. The specialized architecture makes it vulnerable to alterations in its tissue composition. Intramembranous ossification occurs by coordinated processes of production, maturation and mineralization of osteoid matrix [8]. Initially osteoblasts produce a thickening osteoid seam by collagen deposition without mineralization. This is followed by an increase in the mineralization rate and the final stage where collagen synthesis decreases and mineralization continues until the osteoid seam is fully mineralized. As part of the process, mineralization time lag appears to be required for allowing modifications of the osteoid so that it is able to support mineralization [9]. Indeed, fast growing Atlantic salmon has been shown to exhibit low vertebral mineral content and mechanical strength, together with an increased risk of developing vertebral deformities [10,11].

Skeletal growth depends upon the dynamic equilibrium between cartilage production and bone apposition rate [12]. Ontogeny and growth of the vertebral column is under control of regulatory mechanisms involving transcription factors, signaling molecules and extracellular matrix proteins. The pathways of chondrocyte and osteoblast differentiation are interconnected during vertebral formation and must be coordinated. In particular, regulatory proteins, like the transcription factors Sox9, Runx2, Osterix, Twist and Mef2c have distinct functions both in the establishment of the vertebral bodies and later in the differentiation and maturation of specific skeletal cell types (review [13]). Similarly, signaling molecules like bone morphogenetic proteins (Bmp2 and Bmp4), and hedgehog proteins (Ihh and Shh) plays different roles both during cell differentiation and skeletal tissue ontogeny [14-16]. Osteoblasts and chondrocytes secrete the collagen fibers and ground substances of bone and cartilage. These cells are also responsible for the mineralization of the matrix through secretion of specialized molecules, such as Alkaline phosphatase (ALP), Osteocalcin and Osteonectin that binds inorganic minerals [17,18]. A widely accepted view is that the spatial restriction of ECM mineralization to bone is explained by osteoblast-specific gene products that initiate the formation of hydroxyapatite crystals (Ca_10_[PO_4_]^6^[OH]^2^) [19]. The requirement for specifically expressed genes in osteoblasts (e.g. *col1, osteocalcin *and *osteonectin*) and chondrocytes (e.g. *col2 *and *col10*) to initiate the formation of matrix or control the growth of hydroxyapatite crystals is supported by numerous studies [18,20,21]. Furthermore, Matrix metalloproteinases (MMPs) and Tartrate-resistant acid phosphatase (TRAP) are involved in degradation of ECM and in the bone remodeling process performed by the osteoclasts [22].

In this work, 20 skeletal genes were used to study the effect of long term hyperthermic exposure on vertebral development and growth in Atlantic salmon. Fish exposed to high temperature (high intensive regime) had a significant higher incidence of deformities than fish from the same origin reared under a conservative temperature regime (low intensive regime). The study was aimed at exposing differences in risk level between the groups, rather than elaborating the pathologies of deformed vertebrae, hence, the study concentrated on phenotypically normal fish from both temperatures. Significant changes in gene transcription were found between phenotypically normal vertebrae of both groups, including down-regulation of genes encoding proteins important for mineralization. Further, *in situ *hybridization (*ISH*) and histological staining revealed phenotypical and functional changes in the arch centra. Our results are of basic interest for understanding bone metabolism and deformities, as well as a tool for assessing fish welfare in practical farming.

## Results

In the present study we analyzed and compared Atlantic salmon vertebrae from high and low temperature intensity regimes. Rate of development and growth was influenced by temperature regime as observed through SGR and time of sampling. The development from fertilization to first feeding lasted 5 months in the low intensive regime at 6°C, compared to 3 months in the high intensive regime at 10°C. Juveniles of the high intensive group also grew more rapidly after start-feeding than the low intensive group, where the former reached 2 g in 6 weeks after first feeding, 15 g in 3 months and 60 g in 7 months after first feeding, at a rearing temperature of 16°C. In comparison, the low intensive group at rearing temperature of 10°C reached similar sizes in 11 weeks, 5 months and 10 months, respectively. Accordingly, after start-feeding fish from the high intensive temperature regime displayed a higher SGR than the low temperature fish, 2.82 and 1.96 respectively.

### Radiography, morphology and mineral analyses

**Figure 1:**
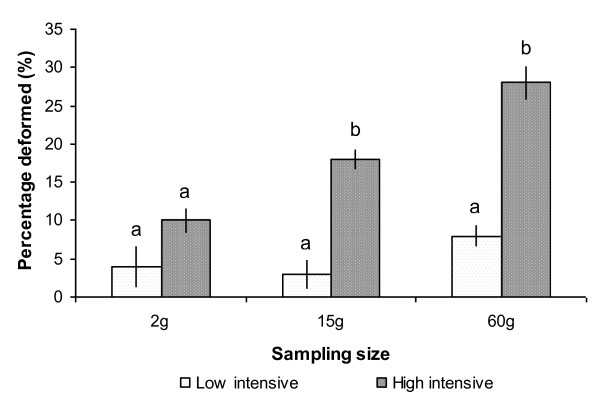
**Frequency (%) of deformities in the vertebral column based on radiographic examination of Atlantic salmon sampled from the low temperature intensive group (white bars) and the high temperature intensive group (black bars) from fertilization until 60 g**. Each bar represents the total number of deformities, scored as present or absent. Data are given in percentage ± st.dev, different letters indicate significant differences (P ≤ 0.01) within the same size group, n = 4 tanks per treatment.

On radiography analysis, the incidence of fish with skeletal abnormalities at 2 g size was 4.0 ± 2.8% and 10.0 ± 1.7% in the low and high intensive groups, respectively (n.s.; not significant). At 15 g size, the difference was more pronounced, 3.4 ± 2.0% and 17.9 ± 1.3% (p < 0.001). At the final sampling at 60 g size, 8 ± 1.4% of the fish in the low intensive group displayed some degree of skeletal pathology compared to 28.1 ± 2.3% in the high intensive group (p < 0.0001), results are shown in figure 1.

**Figure 2:**
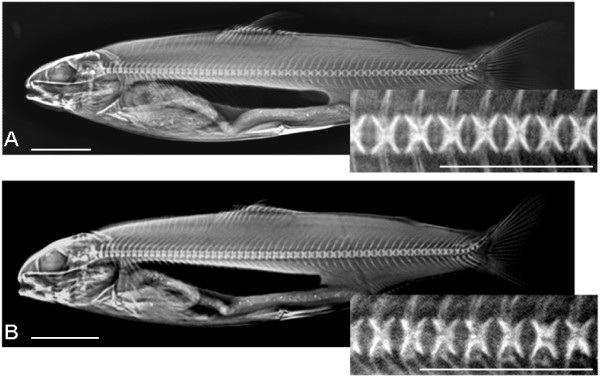
**Radiographic images of Atlantic salmon at 15 g from the low temperature intensive group (A) and the high temperature intensive group (B)**. Scale bar = 1.5 cm. Enlarged picture (to the right) corresponds to the dissected area from underneath the dorsal fin. Scale bar = 1 cm.

Morphometric analyses of vertebral shape demonstrated that fish classified as having a normal phenotype in both groups had more or less regularly shaped vertebrae, but that there was a difference in length-height proportion of vertebrae between fish from the two temperature regimes. Measurements on X-ray images showed that vertebral bodies from the high intensive groups were significantly shorter in craniocaudal direction compared to those from the low intensive groups. The ratios for the high and low intensive group were at 2 g 0.68 ± 0.02 and 0.76 ± 0.02, at 15 g 0.78 ± 0.03 and 0.89 ± 0.06 and at 60 g 0.86 ± 0.01 and 0.94 ± 0.01, respectively (p < 0.001). Examples of vertebral columns with normal phenotype from the high and low intensive group at 15 g are shown in figure 2.

Due to the built-in image contrast enhancement procedures of the semi-digital X-ray system, evaluation of skeletal mineralization as judged by radio density in images was impaired. Nevertheless, a lower contrast in skeletal structures was observed in the high intensity fish, in particular at the 15 g sampling, indicative of a lower mineralization rate at this stage.

Whole body mineral content at the end of the experiment (60 g size) showed low values for Ca, P and Zn content for both temperature regimes (Table 1), with no significant differences between treatments. There was a small, but significant lower level of whole body Fe and Na in the high intensive group. All Fe and Na values were lower than reference values [23], but in correspondence with Ca, P and Zn values, they were within a range which is commonly seen in commercially reared salmon.

**Table 1:** Mineral analysis

	Low intensive group	High intensive group
P	3828 ± 162	3886 ± 285
Ca	3655 ± 341	3700 ± 677
Mg	302.3 ± 8	299 ± 7
Na	1303 ± 70	1084 ± 87*
Fe	9.7 ± 1.2	8.1 ± 0.2*
Mn	0.86 ± 0.05	0.8 ± 0.09
Zn	31.8 ± 2.6	33.5 ± 1.7
Cu	0.8 ± 0.12	0.7 ± 0.09

Mineral content (ppm) in whole body of Atlantic salmon at 60 g from low and high intensive temperature group. Total mineral content was measured in whole fish. Levels were low, but within the range of what are common in commercially reared salmon. Significant numbers (P = 0.05) indicated by *, n = 40 (means ± st.dev).

### Quantitative vertebral mRNA expression

**Figure 3:**
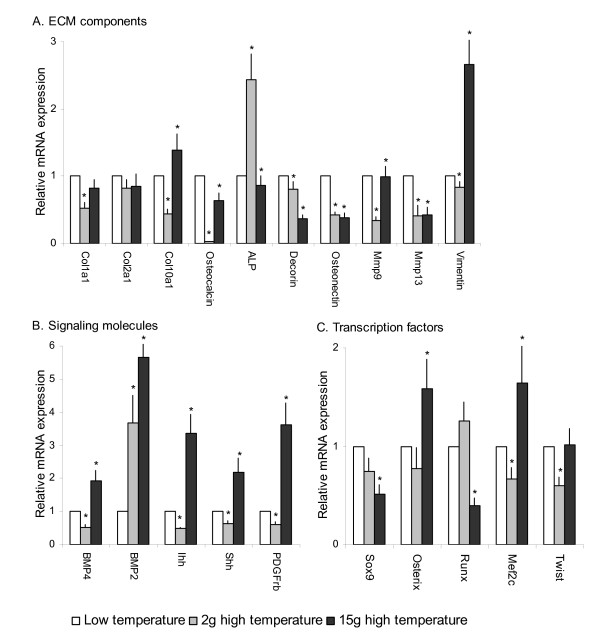
**Relative gene transcription of A. ECM components, B. Signaling molecules and C**. Transcription factors in phenotypical normal spinal columns from 2 g (grey bars) and 15 g (black bars) high temperature intensive group compared to the low temperature intensive group (white bars). Data are given as mean values + SE, n = 15 and significant differences (P = 0.05) are indicated by *. Expression ratios are shown in relative mRNA expression along the y-axis, genes along the x-axis.

The skeletal genes were divided into three groups according to function; ECM constituents, transcription factors, and signaling molecules (Figure 3A-C).

ECM constituents included genes involved in bone matrix production and mineralization and 7 out of 9 of these genes were found to be down-regulated in high intensive group at 2 and 15 g (Figure 3A). Transcription of *col1a1*, *osteocalcin, decorin, osteonectin, mmp9 *and *mmp13 *were reduced in the high intensive group compared to the low intensive group. *Col2a1 *transcription was also down-regulated at both developmental stages, however the values were insignificant. *Osteocalcin *was severely down-regulated in 2 g high intensive group. Converse transcription profiles could be observed for *col10a1 *and *alp *between 2 g and 15 g fish; *col10a1 *was down-regulated at 2 g and up-regulated at 15 g whereas *alp *was up-regulated at 2 g and down-regulated at 15 g.

Temporal changes in transcription factor mRNA expression were found between high and low temperature group, and all genes except *sox9 *showed opposite expression at 2 and 15 g (Figure 3B). In the high intensive group, *sox9 *was down-regulated at 2 g (n.s.) and 15 g, but more pronounced in the latter. Investigation of the two osteoblast markers *runx2 *and *osterix*, revealed opposite mRNA expression levels at 2 and 15 g. *Runx2 *was up-regulated at 2 g (n.s), but down-regulated at 15 g. On the contrary, *osterix *was down-regulated (n.s.) at 2 g, but up-regulated at 15 g. *Mef2c *and *twist *was also down-regulated at 2 g, while up-regulated at 15 g (*twist *n.s.).

Signaling molecules included *bmp2, bmp4, shh *and *ihh*. Expression analysis of mRNA for signaling molecules showed statistically significant differences in expression levels between the temperature regimes and all transcripts were found more abundant in the 15 g group when compared to 2 g vertebrae. *Bmp2 *was the only up-regulated signaling molecule at 2 g, while all signaling genes were up-regulated at 15 g (Figure 3C).

To further examine changes in chondrocyte recruitment and structure between the temperature regimes, we included *platelet derived growth factor receptor b *(*pdgfrb*) and *vimentin*, because of their importance in proliferation and the cytoskeleton, respectively [24,25]. Both transcripts were significantly down-regulated in 2 g, while significantly up-regulated at 15 g (Figure 3A, B).

In summary, we found that out of the 20 genes we analyzed, 8 were down-regulated in both temperature groups (*col1a1, col2a1, osteocalcin*, *sox9, decorin, osteonectin, mmp9 *and *mmp13*), 9 genes were up-regulated in the 15 g high intensive group, but down-regulated at 2 g (*bmp4, col10a1, osterix, ihh, shh, mef2c, twist, vimentin *and *pdgfrb*). And finally, *alp *and *runx2 *were up-regulated at 2 g but down-regulated at 15 g.

### Vertebral tissue morphology and spatial mRNA expression

**Figure 4:**
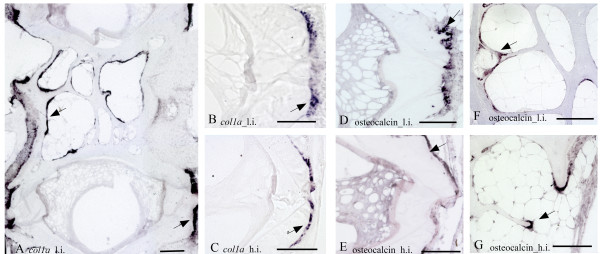
**Transcription of *col1a *(A-C) and *osteocalcin *(D-G) in parasagittal sections of phenotypically normal vertebrae from 15 g salmon reared at low intensive (l.i.) and high intensive (h.i.) temperature intensive regime**. **A**. *Col1a *staining of a large section from l.i. covering 2 vertebrae shows that *col1a *mRNA (arrows) is highly expressed in osteoblasts lining the growth zone on the endbones of the vertebral bodies and the trabeculae. **B**. Higher magnification of the endbones; **C**. The corresponding area in h.i., notice weaker staining. **D**. Osteocalcin expressed in l.i. at the symmetrical growth zones of 2 adjacent vertebrae; **E**. The corresponding area in h.i.; **F**. L.i. Transcription in trabeculae; **G**. The corresponding area in h.i. Arrows indicate positive staining. Ventral side to the left. Scale bar A:200 μm, B-G:100 μm.

**Figure 5:**
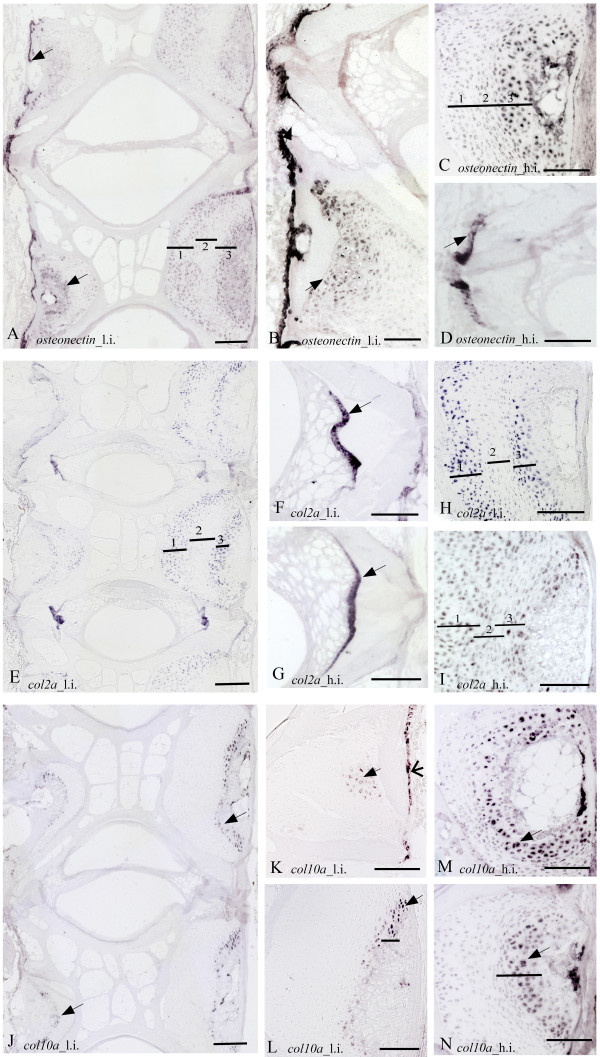
**Transcription of *osteonectin *(A-D), *col2a *(E-I) and *col10a *(J-N) in parasagittal sections of phenotypical normal vertebrae from 15 g salmon reared at low intensive (l.i.) and high intensive (h.i.) temperature intensive regime**. Numbered bars indicate zones of chondrocytes divided into the sub-groups: 1.resting, 2.proliferating and 3.hypertrophic chondrocytes. **A**. *Osteonectin *specific staining of two vertebrae from l.i. revealed staining in zone 1 and 2. **B**. Strong transcription of *osteonectin *is present in osteoblasts lining the growth zone at the endbone and in the hypertrophic chondrocytes (arrow) in l.i. **C**. The arch centra from h.i. Notice increased transcription of *osteonectin*; **D**. Transcription of *osteonectin *in osteoblasts from h.i. appears decreased compared to l.i. **E**. *Col2a *staining of 3 vertebrae from l.i. *Col2a *mRNA localized to chondrocytes in zone 1 and 2. **F**. Higher magnification show distinct staining of *col2a *in the chordoblasts. **G**. Higher magnification of the arch centra with *col2a mRNA *in zone 1 and 2. **H**. In the corresponding area of h.i. a similar, but weaker pattern of *col2a *transcription is visible in the chordoblasts. **I**. Increased temperature lead to a distorted morphology and a more homogenous but weaker staining of *col2a*. **J**. Staining of a large section from l.i. covering 2 vertebrae show that *col10a *mRNA localizes to zone 3 (arrows). **K**. and **L**. Higher magnification reveal *col10a *specific staining in hypertrophic chondrocytes (arrow) and along the rims of the vertebral body (arrowhead). Black bar indicates the narrow zone of transcription restricted to hypertrophic chondrocytes. **M **and **N**. Corresponding area from h.i. show a wider zone of *col10a *positive hypertrophic chondrocytes (black bar). Ventral side to the left. Scale bar A,E,J:200 μm, B-D,F-I,K-N,:100 μm.

In areas where osteoblasts secrete the osteoid matrix, a generally stronger *ISH *signals was apparent in the low intensive group for all probes. The osteogenic marker gene *col1a *showed distinct staining to osteoblasts at the growth zone of the endbones of the vertebral bodies from fish of both temperature regimes (Figure 4A-C). Moreover, *col1a *signal was identified in the bone lining osteoblast cells situated at the lateral surfaces of the trabeculae and along the rims of the vertebral bodies. Investigation of *osteocalcin *mRNA revealed an expression pattern similar to *col1a*, with staining of cells in the osteogenous areas and in bone lining osteoblasts and apical surfaces of the trabeculae (Figure 4D-G). Specifically high *osteocalcin *signal was detected in the proliferative osteoblast growth zones on the endbones of the vertebral bodies. *Osteonectin *mRNA was detected in the osteogenic growth zone of the endbones and lining the exterior part of the vertebral body (Figure 5A-D). The chondrocytic marker *col2a*, hybridized heavily to chordoblasts in the notochord (Figure 5E-G), whereas *col10a *was detected in a continuous layer of cells along the rims of the vertebral body (Figure 5J-L).

**Figure 6:**
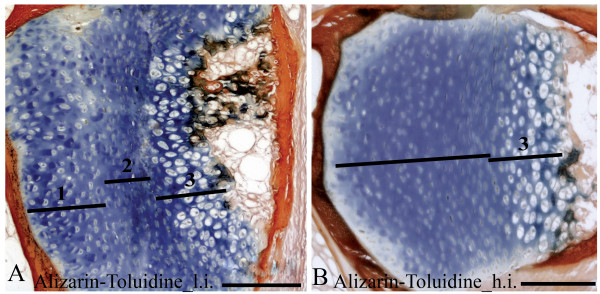
**Parasagittal sections from 15 g salmon vertebrae stained with alizarin red-toluidine blue, showing the arch centra (dorsal side)**. **A**. The chondrocytes localize into bands of 1. resting, 2. proliferating and 3. hypertrophic chondrocytes in fish reared at low temperature. **B**. In comparison, a high temperature regime results in a more uniform cartilage structure without the distinct chondrocytes sub-populations and the hypertrophic chondrocytes appears less calcified. Scale bar: 100 μm.

Alizarin red S and toluidine blue stained chondrocytes in the arch centra and revealed distinct morphological differences between vertebrae from the two temperature groups. The low intensive group was defined by distinct sub-groups of chondrocytes in the different maturational stages i.e. resting, proliferating and hypertrophic. In contrast, the equivalent chondrocytes were more distorted in the high intensive group (Figure 6A, B). *ISH *analysis of *col2a*, *col10a *and *osteonectin *enabled classification of the different chondrocytes into distinct sub-populations of maturational development. *Col2a *hybridized to resting and pre-hypertrophic chondrocytes in two distinct bands of both low and high intensive group, but the mRNA expression was more evenly distributed in all cells of the latter group (Figure 5H, I). There were also generally less proliferating chondrocytes that tended to be less compact in this group. In proliferating chondrocytes we detected strong *col2a *mRNA expression in the high intensive group, but no expression in the low intensive group. Analysis of *col10a *showed restriction to the pre-hypertrophic and hypertrophic chondrocytes located in the deep cartilage zone (Figure 5M, N). *Osteonectin *was also expressed in chondrocytes and the signal increased towards the hypertrophic chondrocytes (Figure 5A-C). The pre-hypertrophic chondrocyte zone was found to be expanded in the high intensive fish and both *col10a1 *and *osteonectin *showed an expanded expression domain corresponding to an increased hypertrophic zone. No signal was detected in any of the samples hybridized with sense probes (data not shown).

**Figure 7:**
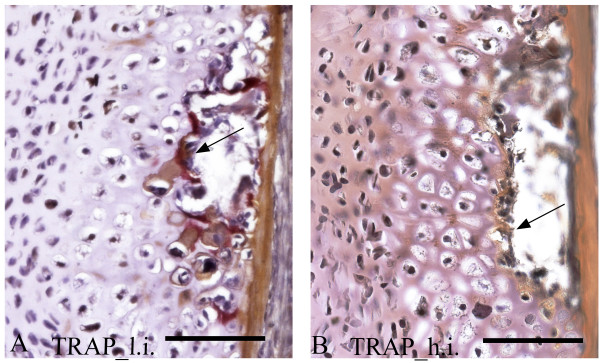
**staining of parasagittal sections from 15 g salmon vertebrae, showing the arch centra (dorsal side)**. **A**. Positive staining (arrow) in the erosive front of cartilage indicating born resorption in neural arches of spinal columns from the low temperature group. **B**. No TRAP activity was found in the corresponding areas (indicated with an arrow) in samples from the high temperature group. Scale bar: 100 μm.

In normal spinal columns from the low intensive group, positive TRAP staining was detected at the ossifying boarders of the hypertrophic chondrocytes in the arch centra. No positive staining was detected in samples from the high intensive group (Figure 7A, B).

## Discussion

The presented study aims at describing the molecular pathology underlying the development of vertebral deformities in Atlantic salmon reared at a high temperature regime that promotes fast growth during the early life stages. Within the period investigated, vertebral bodies form and develop and the skeletal tissue mineralizes. Rearing at high temperatures resulted in higher frequencies of vertebral deformities, as expected. The vertebral pathology observed in this study was most likely induced both during the embryonic development and after start-feeding, since the incidence of deformities continued to increase throughout the experiment after the first radiographic examination at 2 g. Similar temperature regimes before and after start-feeding have independently been shown to induce vertebral defects in juvenile salmon [1,26]. However, whereas high temperatures during embryonic development is commonly related to somitic segmentation failure, deformities later in development may possibly be linked to fast growth induced by elevated temperatures and the impact this might have on the natural maturation and ontogeny of the vertebral bodies [3,10,11]. This causative relation has been shown for fast growing underyearling smolt that has a higher incidence of vertebral deformities than slower growing yearling smolt [27]. Further, morphometric analyses showed that elevated water temperature and faster growth is manifested by a difference in length-height proportion of vertebrae between fish from the two temperature regimes. Similar decrease in length-height proportion was described for the rapid-growing underyearling smolt [27]. Radiographic observations indicated a lower level of mineralization of osteoid tissues in the high temperature fish. However, we could not find any pronounced altered mineral content between the two temperature regimes. The observed values were low compared to reference values [23], but in a range commonly observed in commercially reared salmon. Apparently, whole body mineral analysis seems insufficient to assess problems related to the development of spinal deformities.

To determine whether the difference in likelihood of developing vertebral deformities between the two groups could be traced back to an altered gene transcription, we examined the expression of selected skeletal mRNAs in phenotypical normal salmon fry at 2 and 15 g. Histological examination of 15 g fish was included to improve interpretation of the transcriptional data. The selected genes showed conservation and similar spatial expression with those examined in other vertebrates, supporting that most of the factors and pathways that control skeletal formation are highly conserved in vertebrates.

The lower transcription of ECM genes such as *col1a1*, *osteocalcin*, *osteonectin *and *decorin *suggests a defect in the late maturation of osteoblasts [17,21,28]. The correlation to impaired mineralization is supported by the shorter vertebral bodies in the high intensive groups throughout the study, as well as the impaired mineralization indicated by low contrast observed on X-ray. *Col1a1 *is the primary ECM component secreted by osteoblasts in the trabecular bone and growth plate and defects in the synthesis of *col1 *or type 1 procollagen have been found in several heritable disorders of connective tissue. Likewise, defects in the assembly of Col1 fibrils have been reported to cause abnormally thin and branched structures [29]. Decreased diameter and crosslink density of the collagen fibers have been suggested to reduce thermal stability of collagen and thereby the tissues ability to support load during elevated temperatures [30]. In chum salmon, *Oncorhynchus keta*, the denaturation temperature of collagen type 1 from skin has been reported to be about 19°C [31]. The collagen fibres are further organized and stabilized by a range of non-collagenous proteins, which functions by linking other proteins and minerals to the ECM scaffold. Decorin, which belongs to the small leucine rich repeat proteoglycan (PG) group (SLRPs) is involved in determining the mature collagen fibril structural phenotype and tissue function by facilitating protein-protein interaction with a range of other matrix components (mainly collagen fibres) and with the mineral phase during the formation of calcified tissues [32]. As a result, decorin has been shown to increase tensile strength of the collagen-decorin fiber [33]. Further, osteonectin is a phosphorylated glycoprotein that binds to collagen fibrils, calcium, and hydroxyapatite, linking the bone mineral and collagen phases and perhaps initiating active mineralization in normal skeletal tissue [17,34]. *Osteonectin*-null mice display decreased trabecular bone volume and have bone of lesser stiffness than control mice [35]. *Osteocalcin *mRNA expression also serves as a useful molecular marker of mineralization because it is associated with the maturation of bone cells and mineralization [18,36]. *Alp *is another marker gene for bone cell maturation and mineralization. Inhibition of *alp *activation, by for example heat or by gene knockout, inhibits calcification and causes mineralization defects in cultured bone cells and mice [37]. In addition, mutations in the *alp *gene lead to hypophosphatasia, in which bone matrix formation occurs, but mineralization is inhibited [38]. Our results showed that *alp *was down-regulated in the high intensive 15 g group, but up-regulated in 2 g fish. This may indicate that *alp *is a limiting factor for mineralization after long term exposure to the high temperature regime. Altogether, the simultaneous down-regulation of genes encoding structural proteins taking part in the bone matrix and mineralization strongly supports an assumption that disturbances of these processes constitute an important part of the mechanisms of development of vertebral deformities.

As for the ECM genes involved in osteoblast development and mineralization, high intensive temperature treatment had a significant effect on the transcription of transcription factors and signaling molecules involved in these processes. Intriguingly, Runx2 and Osterix, known as master regulators of osteoblast differentiation [39,40], exhibited opposite mRNA expression levels at 2 and 15 g. *Runx2*-null mice have osteoblast differentiation arrested [41], while *osterix*-null mice embryos have a significant reduction of *col1 *expression and do not express the late osteoblast specific marker osteocalcin [39]. In addition, we analyzed the bHLH transcription factor *twist*. This gene works as a negative regulator of osteoblastogenesis by inhibiting expression of genes downstream of *runx2 *[42]. At 2 g when *osterix *and *twist *was down-regulated while *runx2 *was up-regulated, osteocalcin was heavily down-regulated as was *col1a1*. The mRNA expression pattern was inverted at 15 g. Then *osterix *and *twist *was up-regulated and *runx2 *down-regulated, while *osteocalcin *and *col1a1 *were weakly down-regulated. Linking these results to the pathways involved in osteoblast development, the required simultaneous activation of *osterix *and *runx2 *did not appear at 2 g or at 15 g. However, Osterix function downstream of Runx2 during osteoblast differentiation, but may be regulated by Bmp2 in a Runx2-independent pathway [43]. Bmp2 can induce ectopic bone and cartilage formation in adult vertebrates [44]. Spinella-Jaegle et al [16] found that cooperation between Bmp2 and Shh was necessary to promote a strong induction of the osteoblast marker *alp *in human mesenchymal cell lines. At both 2 and 15 g, *bmp2 *was highly up-regulated in the high intensive group, possibly as a response to the low ECM mRNA expression and under-mineralized tissue. In addition, *osterix *and *shh *was up-regulated at 15 g, as was *bmp4*. Bmp4 treatment has been shown to stimulate new bone formation and is also expressed in osteoblasts prior to formation of mineralized bone nodules [45,46]. However, in comparison to Spinella-Jaegles *in vitro *findings, we did not detect an increase in *alp *mRNA expression. Further, we detected a weaker signal of *osteocalcin *and *osteonectin *in osteoblasts from the *ISH *of the high intensive group at 15 g. Hence, despite the possible attempt of *bmp2 *to restore bone formation and mineralization, there was still lower transcription of ECM components in the high intensive group at 15 g. Summarized, our results may indicate that osteoblast proliferation and mineralization were restrained in the fast growing group.

The percentage of deformities significantly increased in the high intensive group from 2 g till 15 g, while the percentage was stable in the low intensive group. Hence, this period seems to involve important steps for the developmental fate of deformities. Between these two size stages we observed a change in expression pattern, from a downregulated to an upregulated transcription, of 9 genes, where 8 of them are involved in chondrogenesis. This suggested that chondrocytes go through changes in this period that could be important for the development of the observed pathologies.

In vertebrates as mouse and human [47,48], the growth zones of long bones consists of well defined layers of progenitor, proliferative and hypertrophic chondrocytes [49]. These chondrocytes differ in their morphology, proliferation abilities and secretion of ECM components. For example, transcription of *col2a1 *is characteristic for the proliferative state whereas *col10a1 *is restricted to the hypertrophic state [47,50]. *ISH *of these genes revealed that 15 g Atlantic salmon raised at the low intensive regime also had distinct sub-populations of progenitor, proliferative and hypertrophic chondrocytes at the growth zone of the neural and haemal arches. On the contrary, more distorted layers were found in Atlantic salmon raised at the high intensive regime. Moreover, an increased zone of hypertrophic chondrocytes was found in the proximity of the mineralized bone matrix in the high intensive group. Once these hypertrophic chondrocytes are fully differentiated, matrix calcification would normally be initiated [12]. However, we could not identify any variance in mineralization at the ossifying borders of the hypertrophic chondrocytes when examined by histological Alizarin red S staining.

The increased zone of hypertrophic chondrocytes in the high intensive group and the up-regulated transcription of hypertrophic marker genes suggest an arrest prior to the final maturation of chondrocytes. Thus, these chondrocytes seems unable to initiate mineralization. The chondrocyte hypertrophy marker *col10a1 *and its activator *mef2c *[51] were both up-regulated at 15 g in the high intensive group. Moreover, *ihh*, a repressor of terminal hypertrophic differentiation [52], was found to be highly up-regulated, whereas *sox9*, which is involved in early chondrocyte differentiation, and its downstream structural protein *col2a *[53], were down-regulated. The severely down-regulation of *runx2 *at 15 g is of interest, since *runx2*-null mice embryos have a narrow zone of proliferating chondrocytes and a wide zone of hypertrophic chondrocytes [54]. In addition, *bmp4*, which was up-regulated at 15 g, has been shown to accelerate the hypertrophic maturation process [55]. Interestingly, we also found an up-regulated expression of *pdgfrb *mRNA at 15 g. Kieswetter and collaborators [25] have reported that chondrocytes respond to PDGF by enhancing proliferation and cartilage matrix production while maintaining the cells in a less mature phenotype; corroborating our findings that the chondrocytes are some how arrested in the late hypertrophic stage at 15 g with a reduced possibility of completing the endochondral ossification process with calcified bone as end product. Similar findings have also been shown in rat ulnae, where loading was associated with an increased hypertrophic zone in the growth plate [56], but mineralization rate was suppressed [57]. Another interesting comparative pathological condition to our findings in salmon is tibial dyschondroplasia (TD), a metabolic disease of young poultry that affects the growth of bone and cartilage. The lesion is morphologically characterized by an accumulation of chondrocytes that appear to be unable to differentiate past a pre-hypertrophic stage [58]. TD often occurs in broilers and other poultry that have been bred for fast growth rates. The tibial cartilage does not mature enough to ossify, which leaves the growth plate prone to fracture, infection, and deformed bone development.

The observed shorter phenotype of vertebral bodies from the high intensive group might have been a consequence of higher mechanical load in fast growing fish coincidental with a lower transcription of supportive ECM components. Together with the up-regulation of hypertrophic genes in high intensive fish at 15 g, we also found increased transcription of *vimentin*. Vimentin filaments have been shown to regulate the swelling pressure of chondrocytes [59] and strengthen resistance to mechanical stress [60]. Hence, the increased activation of *vimentin *and the increased proportion of hypertrophic chondrocytes in the high intensive temperature group at 15 g may reflect an adaptation to the fast growth by prioritizing maturation of chondrocytes that are more resistant to mechanical stress. At 2 g, however, the reduced level of *vimentin *mRNAs might possibly be linked to the mal-adaptive down-regulation of chondrocytic genes in high intensive group. Indeed, disruption of vimentin filaments has been shown to result in loss of cell contact with the surrounding matrix which may alter the signaling dynamics of the cell and in effect shut down transcriptional events [24].

Mineralizing hypertrophic chondrocytes acquire and express most of the phenotypic characteristics of osteoblasts, including high Alp activity and expression of *osteonectin *and *osteocalcin *[61]. These phenotypic traits shared with osteoblasts may be needed to bring about the final phase of endochondral ossification and replace mineralized cartilage with bone [62]. They may also permit mineralized cartilage to act as bone-like structural tissue and allow for a transition from cartilage to bone. In contrast to the down-regulated transcription of *osteonectin *and *osteocalcin*, as determined by real time qPCR, we observed an increased transcription pattern of these genes in the arch centra in the high intensive group by *ISH*. We also observed a tendency of lower transcription of the same genes in osteoblasts of the high intensive group. However, establishment of a calcifiable matrix requires degradation of some matrix molecules. Endochondral bone formation includes the participation of MMPs, which degrade cartilage matrix and allow vascular invasion [63]. At least two proteases are involved in this process; MMP13 which regulates remodeling of the hypertrophic cartilage matrix and MMP9 which has a role in vascularisation of the growth plate [64,65]. When analyzing these MMPs in salmon vertebral columns, a significant down-regulation of both *mmp9 *and *mmp13 *in the high intensive group at 2 g were observed. At 15 g, *mmp13 *mRNA expression decreased even more, while *mmp9 *was significantly up-regulated. Indeed, MMP13 is known as the dominant collagenase in cartilage and its absence cause delay in endochondral ossification [65]. Further supporting the hypothesis that endochondral ossification was in some way delayed in the spinal columns from the high intensive group, *runx2 *deficiency has been shown to inhibit *mmp *expression [66] and lead to mild disturbances of chondrocyte differentiation, as discussed above. In addition, TRAP activity, essential for completing endochondral ossification [22], was absent in the erosive front of cartilage in neural and heamal arches of spinal columns from the high temperature group.

## Conclusion

The presented results contribute to the understanding of the mechanisms involved in development of temperature-induced vertebral pathology by describing changes in vertebral tissue not yet manifesting pathological deviations. Our results strongly indicate that temperature induced fast growth is severely affecting gene transcription in osteoblasts and chondrocytes, leading to a change in the tissue structure and composition. The data presented here indicate that both production of bone and cartilage were disrupted when promoting fast growth using elevated temperature. It is not unlikely that this disequilibrium is involved in the higher rate of deformities observed in the high intensive group. Importantly, management control of deformities and health in general demands precise tools and knowledge to depict any problem as early as possible in the production line. The defined markers of bone and cartilage cell differentiation and matrix formation can be used to investigate how the progression of skeletogenesis is modulated by a variety of factors. Even though differences in the two experimental groups were undetectable externally, rearing at increased temperatures induced consistent transcriptional changes in several genes that correlated with the higher risk of developing deformities later in ontogeny. Hence, this article reveals the potential use of gene transcription profiling as a prognostic approach in aquaculture.

## Methods

### Experimental design

**Figure 8:**
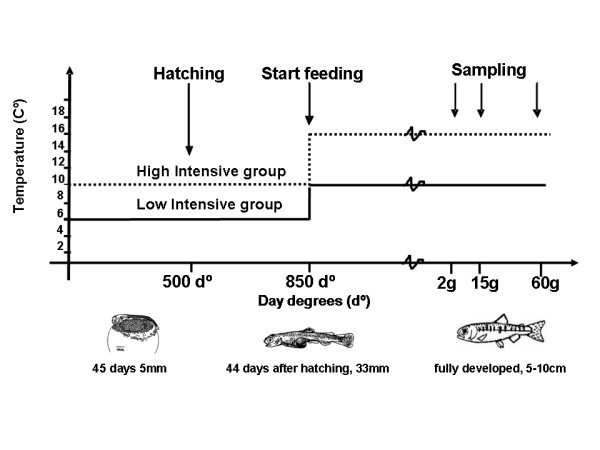
**Experimental overview of the two temperature regimes**. Developmental stage is shown along the x-axis and temperature along the y-axis. The low temperature intensive rearing conditions are shown as a black line and the high temperature intensive conditions as a stippled line. Sampling was conducted at 2 g, 15 g and 60 g. Experiment was terminated when the fish reached 60 g.

The fish experiment was done at Nofima Marine at Sunndalsøra, Norway, in 2007 with Atlantic salmon from the Salmobreed strain. Two experimental temperature regimes were set up; a high intensive temperature group and a low intensive temperature group (Figure 8). Pooled batches of unfertilized eggs and milt were transported on ice to the hatchery and were fertilized, rinsed and disinfected according to standard procedures. The eggs were incubated in a hatchery designed for incubation of small egg volumes, with approximately 0.2 liters of eggs per unit in six units per temperature regime. During egg rearing water supply was continuous from two temperature controlled tanks stabilized at 10 ± 0.3°C and 6 ± 0.3°C, respectively, monitored twice daily. At 850 d° (day degrees = sum of daily temperature), a selection of fry were mixed and transferred to 150 liter tanks for start-feeding, four tanks per temperature regime. The number of fry per tank was 400. Water flow in the tanks was adjusted throughout the experimental period to secure oxygen supply in excess. The fish were fed commercial diets and the light was continuous. The temperature for the high intensive tanks was gradually increased at first feeding to 16 ± 0.3°C and the temperature for the low intensive tanks was gradually increased to 10 ± 0.3°C (1°C per day). These temperatures were kept stable until the average size in each group reached 20 g. At this size, the differentiated temperature treatment was ended. 100 fish per tank were selected randomly, and were tagged individually with pit-tags in the abdominal cavity. Fish from the four tanks on same temperature regime were mixed in a larger tank, and reared at ambient temperature until termination at 60 g. Specific growth rates (SGR) in the period between start-feeding and 60 g were measured according to equation SGR = ((endweight/startweight)^^(1/days)^-1) × 100).

### Tissue sampling, radiography, morphology and mineral analyses

Vertebral columns of phenotypically normal specimens from both temperature groups were sampled for gene expression analysis at 2 and 15 g size and histological analysis at 15 g size. The term phenotypically normal was defined as vertebral columns without any obvious aberrations or deformities when imaged by radiography at sampling. For this purpose, fish were heavily sedated in MS 222 (Tricaine methane sulphate, Pharmaq, Overhalla, Norway) (150 mg/litre) and imaged with an IMS Giotto mammography system (model number 6020/3, IMS Giotto, Bologna, Italy) equipped with a FCR Profect phosphorus film plate (Fuji Medical Inc., Japan). The resulting 20 pixels/mm images were enhanced with digital software (Fuji Computed Radiography Console) and evaluated manually concurrent with sampling. Fish without any specific pathology of the vertebral column were identified for sampling, and killed by an anesthetic overdose. Approximately ~5 vertebral bodies (~1 cm) were carefully dissected from the area under the dorsal fin. For gene expression analyses, samples were flash-frozen in liquid nitrogen and transported on dry-ice to a -80°C freezer for storage. For histological analysis, vertebrae were fixated in 4% PFA for 24 h at 4°C, dehydrated in ethanol (25, 50 and 70%) and stored at 70% ethanol at -20°C. At 2 g size, 350 fish were screened and a total of 40 were sampled for this study. At 15 g size, 900 fish were screened, and 70 were sampled. Fish that were not selected for sampling following radiography were transferred to clean water and returned to the rearing tank. At 60 g size, following an on-growing period on ambient temperatures, 800 fish were radiographed, 100 per original first feeding tank.

Incidence of skeletal deformities was recorded on radiographs from all samplings, and the presence or absence of vertebral pathology was recorded. It should be noted that fish with deviant vertebral morphology, mainly those with fusion type changes, were heavily sampled on basis of live X-ray at 2 g and 15 g (Ytteborg, manuscript in progress). This gives an underestimation of the differences between the two groups. In order to quantify differences observed in proportions of vertebral bodies, length and height of vertebral bodies were measured on X-rays (ImageJ 1.39, NIH, USA), The length (craniocaudal) and height (dorsoventral) of 5 vertebral bodies under the dorsal fin was measured in 12 individuals from each group at 2, 15 g and 60 g, and the length: height ratio was calculated.

At termination of the experiment, fish were sampled for analysis of whole body mineral content. Four samples per treatment were taken, one per each of the original first feeding tanks. Each sample consisted of 10 fish, which were pooled before analysis. The samples were stored frozen at -20°C, and were homogenized prior to analysis. The dry matter of samples was determined after drying at 104°C for 16 h. For mineral analysis, samples were prepared as described [67,68] before analyzed by inductive coupled plasma (ICP) mass-spectroscopy.

### Statistical analyses

A one-way analysis of variance model on incidence of deformities were carried out by SAS 9.1 software (SAS Institute Inc., USA), including the fixed effect of temperature regime. Statistics for gene transcription analysis are described in the real time qPCR section.

### RNA isolation and cDNA synthesis

Tissue homogenization from 15 replicates from each treatment and developmental stage was achieved in a mortar with liquid nitrogen. Total RNA from the powdered vertebrae was isolated by using TRIzol™ and Micro to Midi Kit^® ^(Invitrogen, MD, USA). Samples were treated with DNase1 (Invitrogen) before cDNA synthesis using oligo(dT) and Taqman Gold RT-PCR kit (Applied Biosystems, CA, USA). The cDNA synthesis was performed with 10 min primer incubation at 25°C, 60 min RT step at 48°C and 5 min RT inactivation at 95°C in accordance to the manufacturer's protocol. All reactions were performed in accordance to the manufacturer's protocol.

### Sequence information and primer design

Primers for expression analysis were based on known Atlantic salmon sequences or on conserved regions of known teleost sequences paralogues. Primers were designed using the Vector NTI Advance 10 (Life technologies, MD, USA), and NetPrimer (PREMIER Biosoft, CA, USA) software. All PCR products were cloned using pGEM T-easy (Promega, WI, USA) and sequenced with Big Dye Terminator chemistry and the ABI 3730 automated sequencer, both delivered by Applied Biosystems. The obtained Atlantic salmon sequences were analyzed by BLAST and deposited in the Genbank database (Table 2).

**Table 2:** Primers used for Real time qPCR (RT) and probes for in situ hybridization (ISH)

Gene	Orientation	Genbank	Tm	Use	Sequence (5'-3')
Extracellular Matrix constituents					

Col1a1	Forward	FJ195608	68.6	RT	AGAGAGGAGTCATGGGACCCGT
	Reverse		68.8	RT	GGGTCCTGGAAGTCCCTGGAAT
	Forward		69.8	ISH	TAGCCGTGGTTTCCCTGGTT
	Reverse		68.7	ISH	CCGGGAGGTCCAAATCTACC
Col2a1	Forward	FJ195613	62.6	RT	TGGTCGTTCTGGAGAGACT
	Reverse		62.7	RT	CCTCATGTACCTCAAGGGAT
	Forward		64.4	ISH	GCTGGCGAGACAGGAGAGA
	Reverse		63.4	ISH	GCCTCATCAGCCCTCATGTA
Col10a1	Forward	EG837148	66.4	RT	TGGTGCTCTTTGACTGCCTGTAA
	Reverse		65.1	RT	CATCCTGTGTGTTGCAATATCACA
	Forward		61.9	ISH	AACAAGGGCTTCTTGGATCA
	Reverse		60.9	ISH	CATAATGCATCCTCAGGCAT
ALP	Forward	FJ195609	62.6	RT	CTAGTTTGGGTCGTGGTATGT
	Reverse		62.2	RT	TGAGGGCATTCTTCAAAGTA
Osteocalcin	Forward	FJ195611	62.9	RT	GTGAACCAACAGCAAAGAGA
	Reverse		62.8	RT	CCAGGTCCTTCTTAACAAACA
	Forward		59.6	ISH	CTCATACTTGTTGATCGTCCAG
	Reverse		60.2	ISH	TCTTTCTCTCTCGCTCTCCC
Osteonectin	Forward	FJ195614	65.3	RT	ATTACTGAGGAGGAGCCCATCATT
	Reverse		65.9	RT	CCTCATCCACCTCACACACCTT
	Forward		64.6	ISH	CTGAACGATGAGGGTGTGGA
	Reverse		67.2	ISH	CGAGTGGTGCAGTGCTCCAT
Decorin	Forward	DQ452069	65.0	RT	GAACCTGGCTAAGCTGGGTCTAA
	Reverse		66.0	RT	GAACAGGCTGATGCCAGAGTACAT
MMP9	Forward	CA342769	65.9	RT	AGTCTACGGTAGCAGCAATGAAGGC
	Reverse		65.7	RT	CGTCAAAGGTCTGGTAGGAGCGTAT
MMP13	Forward	DW539943	66.9	RT	TGATGTCCAAGTCAGCCGCTTC
	Reverse		63.6	RT	AAGGAGGCAGGAGGAAGAGG
Vimentin	Forward	GE618371	62.5	RT	CAAGATCCTGTTAGCAGAGCT
	Reverse		67.8	RT	TGGTCTGCCACTTGCGATTGTC

Transcription factors					

Sox9	Forward	EU344852	62.8	RT	CCTGCAAACAAGACAAGGT
	Reverse		62.8	RT	GGGTCGAGTAGATTCATACGA
Runx2	Forward	FJ195615	64.8	RT	CCACCAGGGACAGACACAGAT
	Reverse		64.0	RT	GAACGGACTGAGATCTGACGAA
Osterix	Forward	FJ195612	63.0	RT	TCCCATAGACTTTCCCACA
	Reverse		62.9	RT	TGCCTCAGGACATGTACAA
Mef2c	Forward	GU252207	62.5	RT	CACCGTAACTCGCCTGGTCT
	Reverse		62.3	RT	GCTTGCGGTTGCTGTTCATA
Twist*	Forward	AY546100	61.8	RT	GCTTCAAAAGTGGAGACCGTTT
	Reverse		61.6	RT	GGGAGAACTTGAGCCCTCTTC

Signalling molecules:					

BMP4	Forward	FJ195610	62.2	RT	TCAAGTTGCCCATAGTCAGT
	Reverse		62.6	RT	CACCTGAACTCTACCAACCA
BMP2	Forward	BT059611	62.5	RT	ATGTGGTATTGCACCCATT
	Reverse		62.9	RT	ATGGACAGTTTCCCAATGA
Shh*	Forward	AY370830	61.7	RT	CCGGCTCATGACTCAGAGATG
	Reverse		62.2	RT	TATCCCTGGCCACTGGTTCA
Ihh	Forward	FJ195617	63.2	RT	GGCTCAATCTCCTCTCTCCAAT
	Reverse		63.3	RT	GCTTGGTTGGGAGATATGCA
PDGFrb	Forward	BT071824	62.2	RT	TAGGACCAGCCGATGTTACTGC
	Reverse		62.9	RT	TCTCTGAGCCTCTCGATGT

Housekeeping genes:					

EF1a**	Forward	DQ834870	71.3	RT	CACCACCGGCCATCTGATCTACAA
	Reverse		69.4	RT	TCAGCAGCCTCCTTCTCGAACTTC
GAPDH***	Forward	BT043825.1	72.4	RT	TGGTGCAGAACCTCATGGTCCTCA
	Reverse		70.2	RT	ATCCCGGATGATTCCAAAGTCGTC
Hsp90β ***	Forward	BX074486	59.7	RT	AATGGGTAACCTGGTCAGTG
	Reverse		60.6	RT	GACTCAGGCAAAGGAACCTT

*Wargelius et al. [3] **Olsvik et al. [69] ***Takle et al. [2]

### Real time PCR

Triplicate real-time qPCR reactions were performed using the Light cycler 480 and SYBR Green chemistry (Roche, Switzerland) at the following thermal cycling conditions: 95°C for 10 min, followed by 45 cycles at 95°C for 15 s, 60 ± 1°C for 15 s and 72°C for 15 s. Further, specificity was assessed by the melting curves, determined post PCR (95°C for 15 s, 60°C for 1 min and 97°C continuous). PCR efficiencies for each target and the three housekeeping genes; *elongation factor 1a *(*el1a), heat shock protein 90 β (hsp90β*) and *glyceraldehyde 3*-*phosphate dehydrogenase *(*gapdh*) were tested as endogenous controls. Relative target gene mRNA was normalized to relative *el1a *mRNA levels for all sample, as recommended by Olsvik et al. [69]. The transcription ratios of the 20 genes in all individual vertebrae from the two developmental stages were tested by using the Relative Expression Software Tool, REST, according to Pfaffl et al. [70]. Differences between the transcription ratios were tested for significance by the Pair Wise Fixed Reallocation Randomization Test^© ^[70].

### *In situ *hybridization and histology

Samples of phenotypically normal vertebrae from low and high intensive group at the 15 g developmental stage were analyzed by *ISH *and histological analysis. Samples were dehydrated stepwise for 24 h and clearing carried out in xylene (Merck, Darmstadt, Germany) for 2 × 24 h before embedding in Technovit 9100 (Heraeus Kulzer, GmbH, Wehrheim, Germany), according to the procedure described by Torgersen et al. [71]. Parasagittal serial sections were cut from vertebral columns by using a Microm HM 355S (Thermo Fisher scientific Inc., PA, USA) and mounted on pre-coated slides (0.01% Poly-L-lysine (Sigma) and 2% polyvinyl acetate glue (Casco, Arnheim, Germany)). *ISH *was carried out with digoxigenine labeled (DIG RNA Labeling Kit, Roche) probes as described [71]. A total of five ECM producing genes were analyzed, including *col1a*, *col2a*, *col10a*, *osteocalcin *and *osteonectin*.

Histological examination of vertebrae with toluidine blue and alizarin red S double staining was carried out on deplastified and rehydrated sections. Briefly, the sections were stained for 2-3 min at RT in 0.1% toluidine blue (Sigma-Aldrich) (pH 2.3) and rinsed in distilled H_2_O followed by alizarin red (Sigma-Aldrich) (pH 4.2) staining for 5 min. Prior to microscopy, the stained sections were dehydrated in ethanol and mounted with Cytoseal 60 (Electron Microscopy Science, Fort Washington, PA, USA). Bright field microscopic analyses were performed on a Zeiss Axio Observer equipped with an AxioCam MRc5 camera and AxioVision software (Carl Zeiss Microimaging GmbH, Göttingen, Germany).

Specimens for paraffin embedding were stepwise rehydrated in ethanol (50 and 25%) and decalcified for 7 days in 10% EDTA solution buffered with 0.1 M Tris base (Merck) at pH 7.0. The decalcified specimens were rinsed in PBS and stepwise dehydrated in ethanol (50, 70 and 100%), before being embedded in paraffin. We used 3 paraffin infiltration steps carried out at 60°C for 2 × 2 h and 1 × 3 h. The specimens were embedded in paraffin, stiffened at room temperature and hardened over night at 4°C. 5 μm serial sections were prepared using a Microm HM 355S. Paraffin sections were floated on demineralised water (25°C), mounted on uncoated slides and dried ON at 37°C. Prior to staining the sections were de-waxed with Clear Rite (Richard-Allan, MI, USA), followed by 2× washes in xylene (Merck) for 5 min each. Sections were then rehydrated before rinsed in dH_2_O. To demonstrate TRAP activity, the Acid phosphatase leukocyte kit No. 387 (Sigma-Aldrich) was used and followed according to the manufacturer's protocol; except that incubation lasted for 2 h at 37°C. Subsequently, slides were rinsed in dH_2_O. Specimens were counterstained with Mayers hematoxylin (Sigma-Aldrich) for 30 s and rinsed in running tap water before dehydrated, cleared and mounted with Cytoseal 60 (Electron Microscopy Science). Controls were incubated without substrate.

## Abbreviations

ALP: alkaline phosphatase; BCIP/NBT: 5-bromo-4-chloro-3 indolyl phosphate *p*-toluidine salt/nitro blue tetrazolium chloride; bHLH: basic helix loop helix; BMP: bone morphogenetic proteins; dl: deciliter, Gla: y-carboxyglutamic acid; Ihh: indian hedge hog; MMP: matrix metalloproteinase; PBS: phosphate-buffered saline; PDGFrb: platelet derived growth factor receptor b; PFA: paraformaldehyde; Runx2: runt related transcription factor 2; shh: Sonic hedge hog; Sox9: sex determining region Y-box 9; TRAP: Tartrate-resistant acid phosphatase.

## Authors' contributions

EY carried out the molecular studies, participated in sampling and drafted the manuscript. GB carried out the radiological diagnostics, accompanied in statistical analysis and participated in the experimental design. KH radiographed the fish and participated in the diagnostics and statistical analysis. JT participated in the design of the *ISH *experiments, sequence alignment and probe design, microscopic analyzes and acquisition and interpretation of data. HT conceived the study and its experimental and molecular design, coordinated the sampling, and participated in acquisition and interpretation of data and in the drafting of the manuscript. All authors read and approved the final manuscript.
